# SPVec: A Word2vec-Inspired Feature Representation Method for Drug-Target Interaction Prediction

**DOI:** 10.3389/fchem.2019.00895

**Published:** 2020-01-10

**Authors:** Yu-Fang Zhang, Xiangeng Wang, Aman Chandra Kaushik, Yanyi Chu, Xiaoqi Shan, Ming-Zhu Zhao, Qin Xu, Dong-Qing Wei

**Affiliations:** ^1^State Key Laboratory of Microbial Metabolism, and SJTU-Yale Joint Center for Biostatistics and Data Science, School of Life Sciences and Biotechnology, and Joint Laboratory of International Cooperation in Metabolic and Developmental Sciences, Ministry of Education, Shanghai Jiao Tong University, Shanghai, China; ^2^Wuxi School of Medicine, Jiangnan University, Wuxi, China; ^3^Instrumental Analysis Center, Shanghai Jiao Tong University, Shanghai, China; ^4^Peng Cheng Laboratory, Shenzhen, China

**Keywords:** drug-target interaction, representation learning, Word2vec, machine learning, feature embedding

## Abstract

Drug discovery is an academical and commercial process of global importance. Accurate identification of drug-target interactions (DTIs) can significantly facilitate the drug discovery process. Compared to the costly, labor-intensive and time-consuming experimental methods, machine learning (ML) plays an ever-increasingly important role in effective, efficient and high-throughput identification of DTIs. However, upstream feature extraction methods require tremendous human resources and expert insights, which limits the application of ML approaches. Inspired by the unsupervised representation learning methods like Word2vec, we here proposed SPVec, a novel way to automatically represent raw data such as SMILES strings and protein sequences into continuous, information-rich and lower-dimensional vectors, so as to avoid the sparseness and bit collisions from the cumbersomely manually extracted features. Visualization of SPVec nicely illustrated that the similar compounds or proteins occupy similar vector space, which indicated that SPVec not only encodes compound substructures or protein sequences efficiently, but also implicitly reveals some important biophysical and biochemical patterns. Compared with manually-designed features like MACCS fingerprints and amino acid composition (AAC), SPVec showed better performance with several state-of-art machine learning classifiers such as Gradient Boosting Decision Tree, Random Forest and Deep Neural Network on BindingDB. The performance and robustness of SPVec were also confirmed on independent test sets obtained from DrugBank database. Also, based on the whole DrugBank dataset, we predicted the possibilities of all unlabeled DTIs, where two of the top five predicted novel DTIs were supported by external evidences. These results indicated that SPVec can provide an effective and efficient way to discover reliable DTIs, which would be beneficial for drug reprofiling.

## Introduction

Drug discovery is an issue of global importance, both academically and commercially. Generally, drugs have interactions with specific molecular targets, which are known as drug-target interactions (DTIs). Accurate identification of DTIs can significantly facilitate the processes of drug discovery. Thus, modern drug development calls for more effective and efficient techniques to identify true DTIs from the vast libraries of chemical compounds and protein targets. Numerous efforts have been poured into predictions of DTIs. However, it is still challenging to identify new drugs and their corresponding targets because of the limited knowledge about complex relationships between chemical space and proteomics space. Since *in vivo* and *in vitro* testings are rather costly and time-consuming (Kuruvilla et al., 2002; Haggarty et al., 2003; Valentin et al., 2018), scientists' focus moves more than ever to *in silico* techniques predict potential drug-target associations on a large scale, in which machine learning (ML) is one of the most attractive approaches.

Various machine learning methods have been developed in the last decades, in which the most widely used models are binary classifiers like Random Forest (RF) (Ho, 1998), Support Vector Machine (SVM) (Cortes and Vapnik, 1995), Deep Neural Network (DNN) (Liu et al., 2017), Gradient Boosting Decision Tree (GBDT) (Friedman, 2001), and so on. The performance of machine learning methods relies heavily on data representation (or features). Therefore, the design of data preprocessing and data transformation is of great concern to ensure that the data representation can support efficient machine learning algorithms. Numeric methods have been proposed to excavate drug and target features from their chemical structures and genomic sequences, respectively, such as fingerprints (Morgan, 1965; Ewing et al., 2006) and other molecular descriptors (Van Aalten et al., 1996; Hong et al., 2008) for drugs, amino acid composition (AAC) (Nakashima and Nishikawa, 1994) and physico-chemical properties (Cai et al., 2002) of target proteins, and so on. For example, Nascimento et al. (2016) used “normalized Smith-Waterman, mismatch and spectrum kernels” for the target protein sequences and “the spectrum, Lambda-k, Marginalized, MinMax, and Tanimoto kernels” for the drug's chemical structure to predict DTIs. In the work by Nanni et al. (2014), the drugs were represented by FP2 fingerprints and the representations on the targets were based on autocovariance, entropy, discrete wavelet, and substitution, and so on. The representation of the drug-target pairs was done by concatenating the target descriptors with the FP2 fingerprints of the drug. In the works by He et al. (2010), multiple chemical functional groups for drug-related features and pseudo AAC for protein-related features were extracted to describe drug-target pairs. Chen et al. (2012) integrated protein-protein similarity network, drug-drug similarity network, and known drug-target interaction networks into a heterogeneous network, and then implemented the random walk algorithm on this heterogeneous network for the prediction of DTIs. Rayhan et al. (2017) exploited their algorithms using both structural and evolutionary information to generate informative features. Based on these traditional features, the performance of machine learning algorithms for predictions of DTIs have been gradually improved to a quite high level. However, these feature extraction methods require tremendous manpower and expert insights, and the effectiveness of these features also requires tremendous computations to be proved. Tedious processes of “feature engineering” have to be done before these features can be fed into downstream ML models. In order to facilitate the application of machine learning technologies, it is necessary to make them less dependent on feature engineering.

Representation learning (RL) (Bengio et al., 2013) is a way to introduce artificial intelligence (AI) and prior knowledge to automatically learn continuous, information-rich and lower-dimensional vectors from raw data that can be easily and directly used in ML models. An RL algorithm attempts to discover the latent features that describe the structure of a dataset under certain (either explicit or implicit) assumptions. Nowadays, RL has shown an influential role in effectively extracting features and solving the problem in computer vision, pattern recognition and natural language processing (NLP) (Mikolov et al., 2013a; Sharif Razavian et al., 2014). RL aims to automatically learn the representations (or features) from raw data that can be effectively utilized by downstream machine learning models to improve the performance of the model. Word2vec (Mikolov et al., 2013b) is one of the most popular RL methods, making NLP problems easier to tackle. Inspired by the distributed hypotheses that words found in similar environments usually have similar meanings, the Word2vec model predicts the center word based on its neighbor words in the window of a given size. This method simultaneously learn several language concepts such as Collobert and Weston (2008): (1) the meaning of the word; (2) how words are combined to form a concept (i.e., grammar); (3) how a concept relates to the task. Word2vec effectively removes word-meaning extraction subtasks by providing pre-trained word embeddings for learning algorithms. The word representations computed using Word2vec are very interesting because the learned vectors explicitly encode many linguistic regularities and patterns. Somewhat surprisingly, many of these patterns can be represented as linear translations. For example, the vector of “Paris” minus the vector of “France” plus the vector of “Italy” is very close to “Rome.” In addition to its original utility as a word-embedding method, some of its ideas are effective in sequential data of non-language tasks (Jaeger et al., 2018; Zhang et al., 2019).

Recently, RL brought several breakthroughs in compound space and protein space. Convolutional neural networks were successfully applied on molecular graphs (Kearnes et al., 2016; Coley et al., 2017) and depictions of molecules (Goh et al., 2017b). Latent semantic structure indexing (LaSSI) (Schneider et al., 2017) techniques were adopted to compute chemical similarity from molecular descriptors. Word2vec (Asgari and Mofrad, 2015) has been adapted to protein sequences (ProtVec) for classification of protein families and predication of disordered proteins. Wan and Zeng (2016) used term frequency-inverse document frequency (tf-idf) to learn compound representations from Morgan fingerprints. While substructures of a molecule are hashed to a binary fingerprint (possibly sparse) in the case of the fingerprints, the Mol2vec approach, proposed by Jaeger et al. (2018), forms a vector with continuous and dense values. The SMILES2Vec (Goh et al., 2017a) a model introduces a direct conversion of chemical structures from SMILES (Simplified Molecular-Input Line-Entry System) strings into vectors. These works show that RL technologies represented by Word2vec can automatic learn low-dimensional features from compound and protein feature space and achieve excellent performances, suggesting its advantages in both efficiency and effectiveness.

**Figure 1:**
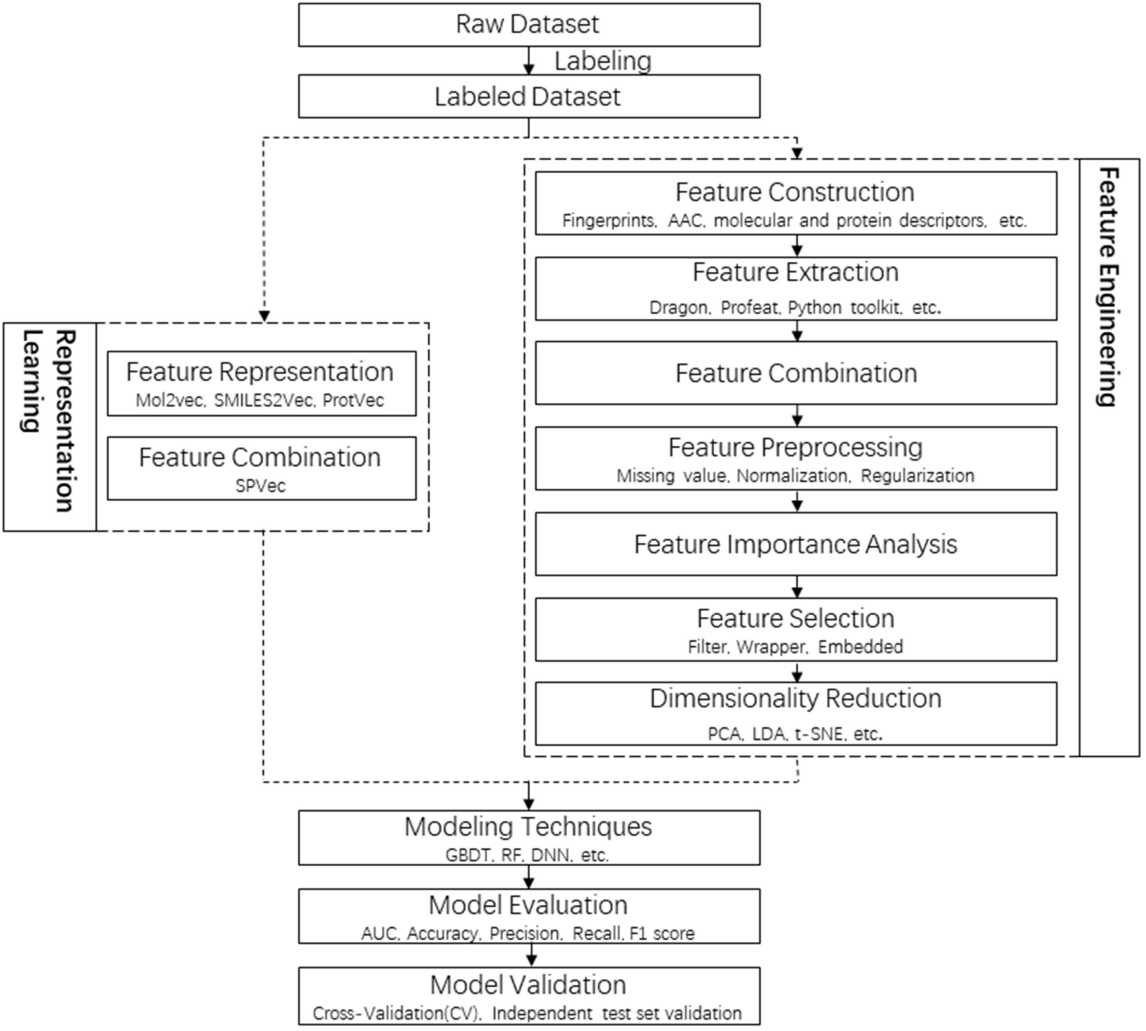
Flowchart of the whole pipeline for DTI prediction in this article **(left)** in comparison to the traditional pipeline **(right)**, with the procedures of feature representations squared in dashed lines.

In this study, new SPVec vectors were constructed via the combination of SMILES2Vec and ProtVec to represent specific DTIs, where the drug representation was simplified by using SMILES directly. The whole pipeline of DTI prediction in this article is shown in Figure 1, in comparison with a tradition pipeline. Not like RL who can atomically learn lower-dimensional features without human resources and expert insights, traditional feature engineering usually contains a lot of steps, including feature extraction, feature selection and dimensionality reduction, while every step need professional knowledge and extra time. For example, Fingerprint-based features are sparse and high-dimensional, thus dimensional reduction is necessary. Feature importance analysis and feature selection might be indispensable for mixed features, such as physicochemical properties, structural and evolutionary information and interaction information. It only takes several hours for feature presentation by SPVec training on a modern quad-core CPU, while dozens of days are required for traditional feature engineering. To evaluate the performance of SPVec, the constructed vectors was fed into several state-of-art machine learning classifiers such as GBDT, RF and DNN on BindingDB (Gilson et al., 2016). The performance and robustness of SPVec were also confirmed by an external validation using DrugBank database. Also, we predicted the possibilities of all unlabeled DTIs in DrugBank database (Law et al., 2014), where two of the top five predicted novel DTIs were supported by external evidences. The results indicated that SPVec can discover reliable DTIs, which could be beneficial for drug reprofiling.

## Method

### Datasets

BindingDB is a public, web-accessible database of measured binding affinities, focusing chiefly on the interactions of target proteins with small, drug-like molecules, was utilized to evaluate the performance of SPVec. The whole BindingDB claims to contain 1,756,093 binding data for 7,371 protein targets and 780,240 small molecules (updated on 2019-05-01). Considering the validity of the features represented, inorganic compounds and protein targets with sequence identity > 75% were removed. In addition, considering the druggability, we excluded interactions with IC50 value missing or >300 nM. Finally we got 36,014 small molecular drugs and 2,099 targets from BindingDB, which may generate over 75 million DTI pairs. Among them, 83,676 pairs are known as positive DTIs, and the rest are undetermined and treated as unlabeled data, from which 83,676 drug-target pairs were randomly selected as a negative dataset.

To further validate our model, we also collected data of DTIs from DrugBank. The data of drugs, targets and their interactions were separated by the date April 20, 2016, with those before it regarded as old while those after it regarded as new. In this way, we constructed five positive datasets as shown in Table 1: (1) dataset_1 consists of all old drugs, old targets and their old interaction pairs; (2) dataset_2 consists of all old drugs, old targets and their new interaction pairs; (3) dataset_3 consists of all new drugs, old targets and their interaction pairs; (4) dataset_4 consists of all old drugs, new targets and their interaction pairs; (5) dataset_5 consists of all new drugs, new targets and their interaction pairs. The largest dataset_1 with all old data was used for model training, while the other four datasets with new data were used to validate the robustness of the models. The generation of corresponding negative datasets of these five datasets are same as that from Binding DB, except that the unlabeled data pool of dataset_2 is the rest of positive interactions of dataset_1 and dataset_2 (6068×3839-14534-3348).

**Table 1:** Number of entries of the five different datasets obtained from DrugBank dataset.

**Datasets**	**Dataset_1**	**Dataset_2**	**Dataset_3**	**Dataset_4**	**Dataset_5**
Drug	6,068	6,068	537	6,068	537
Target	3,839	3,839	3,839	160	160
Interactions	15,434	3,348	1,735	264	37

### Feature Representations

SPVec is a Word2vec-inspired technique to represent latent features of small compounds and target proteins. Word2vec refers to the method that for any word w in dictionary D, specify a fixed length of the real value vector *V* (w) ∈ ℝ^m^, where *V* (w) is called the word vector of w and *m* is the length of the word vector. All of these vectors form a word vector space, and each vector can be regarded as a point in the space. The lexical or semantic similarity between them can be judged by the “distance” between the points.

In particular, we mainly used the Skip-gram model implemented with the Negative-sampling (NEG) method to train the Word2vec-like models. The classical Skip-gram model consists of three layers: the input layer, the projection layer, and the output layer. Take a sample (*w, Context* (*w*)) for example, assuming that *Context* (*w*) consists of *c* words before and after *w*, then a brief description of these three layers is as follows: the input layer is the word vector *V* (w) ∈ ℝ^m^ of the current sample; the projection layer is identity projection, which means projecting *V* (w) to *V* (w); the output layer is a binary Huffman tree, which takes every word appearing in the corpus as the leaf node and frequency of the word as weight. In the revised Skip-gram model here, the negative samples were generated by relatively simple random NEG method instead of Huffman trees, so as to improve training speed and improve the quality of the resulting word vectors. Given that a negative sample subset *NEG*(w) ≠ ∅ for w and $\forall \tilde{w}\in D$, we define $L^{w}\left(\tilde{w}\right)$ as the label of word w, where the label of a positive sample is 1, and that of a negative sample is 0. For a given sample (*w, Context* (*w*)), we want to maximize the following function:

(1) $$\begin{array}{r} g\left(w\right)=\underset{\overset{\sim }{w}\in Context\left(w\right)}{\prod }\underset{w\in \left\{u\right\}\cup NEG^{\tilde{w}}\left(w\right)}{\prod }p\left(u|\tilde{w}\right) \end{array}$$

where

(2) $$\begin{array}{r} p\left(u|\tilde{w}\right)={\left[\sigma \left({V\left(\tilde{w}\right)}^{T}\theta^{u}\right)\right]}^{L^{w}\left(u\right)}\times {\left[1-\sigma \left({V\left(\tilde{w}\right)}^{T}\theta^{u}\right)\right]}^{1-L^{w}\left(u\right)}, \end{array}$$

here $NEG^{\tilde{w}}\left(w\right)$ is a generated subset of negative samples when processing words $\tilde{w}$. For a given corpus *C*, the final objective function is:

(3) $$\begin{array}{r} L=\log G=\log\underset{w\in C}{\prod }g\left(w\right). \end{array}$$

Maximizing this objective function can be performed using the stochastic gradient descent technique.

The same principles in the work by Wan and Zeng (2016) were followed to choose the hyperparameters of Skip-gram. That is, the embedding dimension was set as *d* = 100, the context window size was set as *c* = 12, and the number of negative examples was set as *k* = 15. Using this revised Skip-gram model, SMILES2Vec and ProtVec models were trained for feature representations of drug compounds and target proteins, respectively, and combined into SPVec to represent their interactions. Different from the works by Jaeger et al. (2018), we directly use SMILES of drug molecules rather than Morgan fingerprints as “sentences” to learn the representations, as SMILES strings are more like “sentences” and don't need additional calculations. At the same time, the protocol of Asgari and Mofrad (2015) was followed in training of ProtVec here, where protein sequences were regarded as “sentences” and every three non-overlapping amino acids were regarded as a “word.”

To benchmark our SPVec approach against classical feature extraction approaches, we also extracted manually-designed features of chemical structures and protein sequences. For the ligands, we adopted the MACCS fingerprint (Corey and Wipke, 1969), one of the most widely used “structural fingerprints” based on pre-defined chemical substructures and finally got 166-dimensional compound feature vectors. At the same time, we considered the 20-dimensional AAC as protein descriptors, which were computed via PROFEAT (Zhang et al., 2016), a web server for computing commonly used protein features from their amino acid sequences.

The SPVec features compose a 100-dimensional space in such a way that similar objects are modeled into nearby points. To explore biochemical implications from SPVec features, the feature vectors of small molecular drugs and protein targets in DrugBank are projected from this 100-dimensional space into a 3D or 2D space for easier visualization using t-Distributed Stochastic Neighbor Embedding (t-SNE) (Der Maaten and Hinton, 2008), which is a non-linear dimensionality reduction technique for visualization of high dimensional data in a low-dimensional space, generally in two or three dimensions.

### Machine Learning Models

The feature embeddings learned by SPVec model were then fed into various machine learning models to predict the likelihood of their interactions. The performance in DTIs prediction by three state-of-art machine learning methods RF, GBDT, and DNN was used to evaluate the utility of SPVec embeddings. RF is an ensemble method that combines the probabilistic predictions of a number of decision tree-based classifiers to improve the generalization ability over a single estimator. GBDT is a machine learning technique for regression and classification problems, which produces a prediction model in the form of an ensemble of weak prediction models, typically decision trees. It builds the model in a stage-wise fashion as other boosting methods do, and it generalizes them by allowing optimization of an arbitrary differentiable loss function. DNN is a supervised learning algorithm which could learn non-linear models. It has one or more non-linear hidden layers between the input and output. For each hidden layer, different numbers of hidden neurons can be assigned. Each hidden neuron yields a weighted linear summation of the values from the previous layer, and the non-linear activation function is followed. The weights are learned through backpropagation algorithm or variations upon it. All these models were implemented by Python v3.6 and scikit-learn library (Pedregosa et al., 2011). All the datasets and source codes, as well as a Python module for user-friendly application of the SPVec method are available at https://github.com/dqwei-lab/SPVec.

## Results and Discussion

### Biochemical Implications of SPVec Features

**Figure 2:**
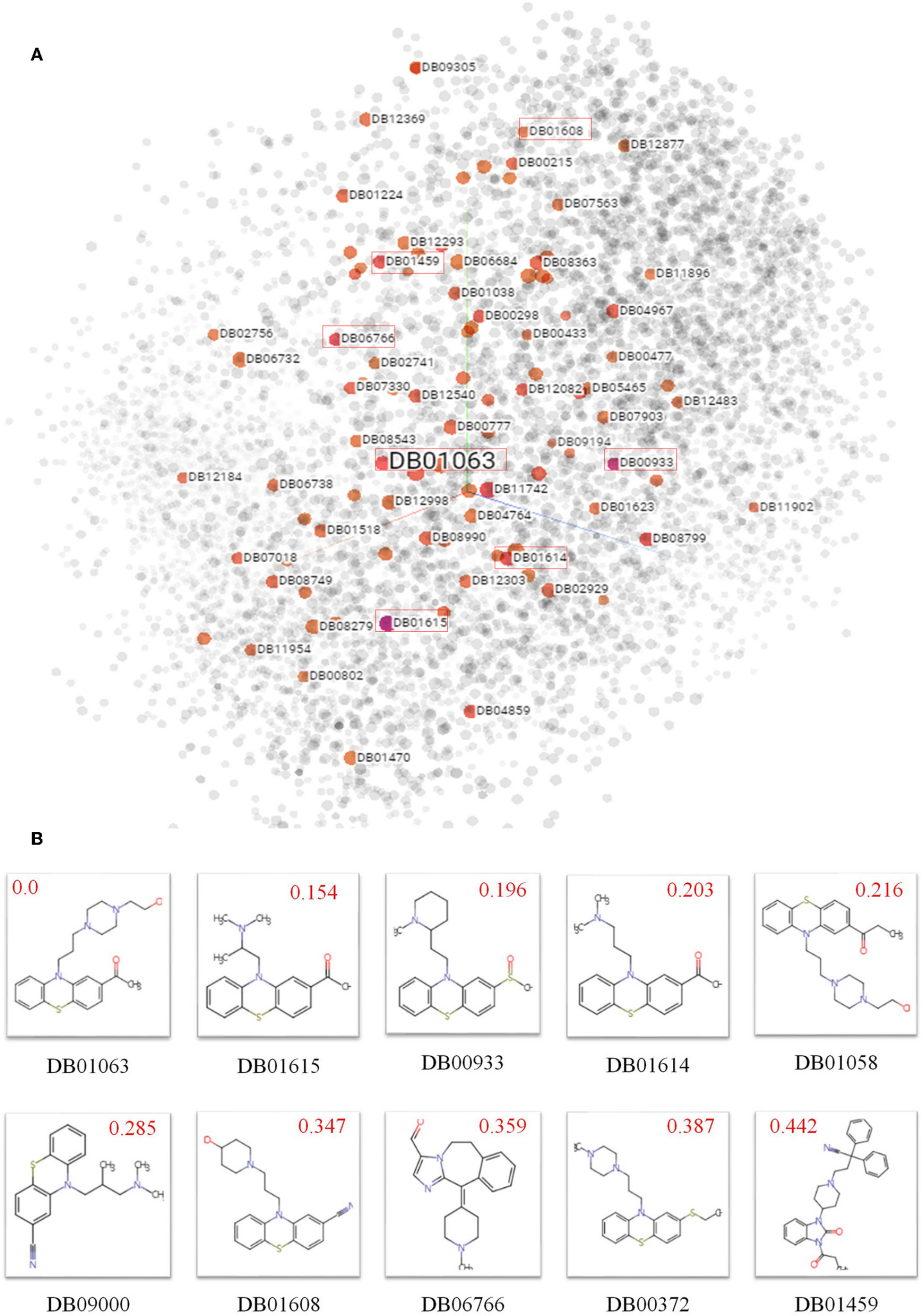
Biochemical implications from SMILES2Vec features. **(A)** Visualizations of the SMILES2Vec vector space of drugs in DrugBank using t-SNE. **(B)** The top 10 drugs most similar to Acetophenazine (DrugBank ID: DB01063) according to their SMILES2Vec vectors. Red values show their cosine distances with Acetophenazine. The smaller the value, the more similar in the chemical structures.

To explore biochemical implications from SPVec features, small molecular drugs in DrugBank are represented into vectors by SMILES2Vec and projected from a 100-dimensional space to a 3D space by t-SNE, shown in Figure 2A, where each point represents a small drug molecule. Since the SMILES2Vec vectors are sums of substructure vectors, they may implicitly capture substructure importance via the vector weight, thus the drugs closer to each other may have more structural and functional similarities. For example, the boxed points stand for part of the top 10 chemicals (because some are masked by other points) similar to Acetophenazine (Drug ID: DB01063), an antipsychotic drug of moderate-potency used in the treatment of disorganized, psychotic thinking and false perceptions. Figure 2B shows the molecular structures of these top 10 similar chemicals, most of which have the phenthiazine substructure (a Sulfur atom and a Nitrogen atom connected with two benzene rings) with neuroleptic and anti-histamine properties. Some of them share the same target. For example, Acepromazine (DrugBank ID: DB01614), Thiethylperazine (DrugBank ID: DB00372) and Acetophenazine have two common targets, D(2) dopamine receptor and D(1A) dopamine receptor. Periciazine (DrugBank ID: DB01608) and Acetophenazine have two common targets, D (1A) dopamine receptor and Androgen receptor). Oppositely, Ceftibuten (DrugBank ID: DB01459), which is relatively far from Acetophenazine, has no Phenthiazine substructure. And in terms of functionalities, Ceftibuten is typically used to treat acute bacterial exacerbations of chronic bronchitis (ABECB), acute bacterial otitis media, pharyngitis, and tonsillitis, which is different from Acepromazine either. Obviously, molecules with similar functional groups are close in the generated SMILES2Vec vector space.

**Figure 3:**
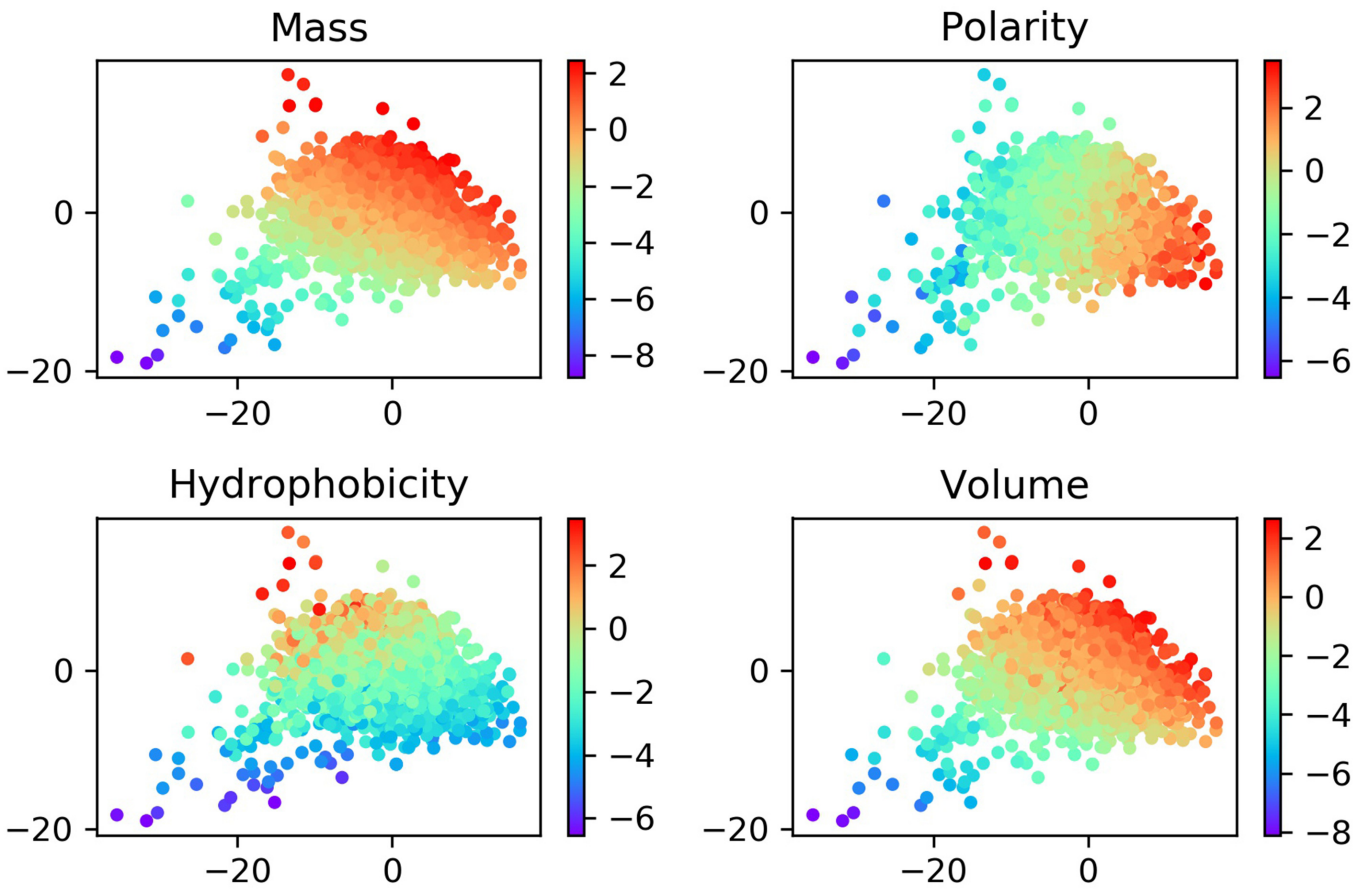
Normalized distributions of biochemical and biophysical properties in a 2D space projected by t-SNE from the 100-dimensional ProtVec protein-space. In these plots, each point represents a protein, and the colors indicate the scale for each property.

Although the ProtVec is only trained based on the primary sequences of proteins, it shows some important biochemical and biophysical implications (Asgari and Mofrad, 2015). In order to study these features, we visualized the distribution of ProtVec vectors by mass, volume, polarity, hydrophobicity. In Figure 3, each point represents a protein, with a color according to its scale in each property. The distribution of these points turns out that proteins with similar biochemical and biophysical properties tend to be closer. This observation indicates that not only encodes protein sequences effectively and efficiently, the ProtVec also implicitly reveals some important biophysical and biochemical patterns of the protein, while AAC only contains information of protein compositions (Nakashima and Nishikawa, 1994).

### Performance of SPVec in Comparison With Traditional Feature Representation Methods

We tested the performance of DTIs predictions for SPVec combined with three state-of-art ML classifiers (GBDT, RF, and DNN) using BindingDB, and compared with the combination of MACCS and AAC features as a baseline. To validate the performance of SMILES2Vec and ProtVec independently, we also constructed another two feature combinations, that is, MACCS-ProtVec and SMILES2Vec-AAC. A summary of classification performances of these four feature combinations on the BindingDB dataset are shown in Table 2, with their ROC curves shown in Figure S1. It is obvious that DTIs predictions based on SPVec vectors are relatively improved than those on the classical feature combination (i.e., MACCS-AAC) when using any of the ML classifiers. For predictions by the GBDT, RF, and DNN classifiers, the AUCs using SMILES2Vec-ProtVec are 13.35, 15.67, and 11.66% higher than MACCS-AAC, respectively. When only molecules are characterized via SMILES2Vec, the AUCs of SMILES2Vec-ProtVec are about 8.86%, 11.57%, and 9.09% higher than SMILES2Vec-AAC. And when molecules are characterized via MACCS, the AUCs of MACCS-ProtVec were about 8.91, 9.42, and 6.85% higher than MACCS-AAC. Therefore, in DTIs predictions single feature representations by ProtVec or SMILES2Vec also partly improve the classification performances. It is also reasonable to expect their individual performances in other tasks related to only drugs or proteins, such as compound property predictions and protein classifications. Table 2 also indicates that features represented by SPVec are quite reliable with different ML models. Based on the datasets from BindingDB, the GBDT, RF, and DNN models resulted in no important difference for classification tasks of DTIs, and all achieved similarly higher AUC score, accuracy, precision, recall, and F1-score.

**Table 2:** Results of classification performance of four feature combinations using three classifiers on BindingDB via 10 × 5-fold cross-validation, with the highest scores highlighted in the bold font.

**Feature combinations**	**Model**	**AUC**	**Accuracy**	**Precision**	**Recall**	**F1-score**
SPVec (SMILES2Vec-ProtVec)	GBDT	0.9923	**0.9680**	0.9695	**0.9667**	**0.9681**
	RF	**0.9927**	0.9675	**0.9808**	0.9540	0.9672
	DNN	0.9617	0.9332	0.9287	0.9248	0.9197
SMILES2Vec-AAC	GBDT	**0.9037**	**0.8272**	**0.8563**	**0.7873**	**0.8204**
	RF	0.8770	0.7974	0.8657	0.7050	0.7772
	DNN	0.8708	0.8124	0.7993	0.7879	0.7126
MACCS-ProtVec	GBDT	**0.9479**	**0.8810**	**0.8908**	**0.8690**	**0.8798**
	RF	0.9302	0.8542	0.8712	0.8322	0.8512
	DNN	0.9136	0.8034	0.8025	0.8097	0.8074
MACCS-AAC	GBDT	**0.8588**	**0.7811**	**0.8077**	**0.7392**	**0.7719**
	RF	0.8360	0.7468	0.8366	0.6150	0.7089
	DNN	0.8451	0.7832	0.7884	0.7726	0.7724

The performance of SPVec based DTIs predictions was also compared with earlier results using different popular classical features or modeling methods, as summarized in Table 3. Compared with these classical features, the features represented by SPVec are much lower in dimensions, which masterly avoid the “Curse of Dimensionality,” and enable ML models to achieve better performances. Especially when some kinds of features are hard to obtain, such as the 3D molecular and protein descriptors, the advantages of SPVec is more evident. It's worth to note that You et al. (2019) and Yu et al. (2012) used DrugBank database with different versions (released on 14 Nov. 2017 and 1 June 2011, respectively), while the datasets for the other predictions (Ezzat et al., 2016, 2017) were from the version 4.3 of DrugBank database (released on 17 Nov. 2015) in which there are 12,674 drug-target interactions between 5,877 drugs and their 3,348 protein interaction partners in total. However, as shown in Table S1, the performances of SPVec did not change a lot using the different versions of database. AUC of DNN, GBDT and RF only increased by 0.0315, 0.0039, and 0.0088, respectively. Therefore, the better performance of SPVec compared with earlier results in Table 3 is still guaranteed.

**Table 3:** AUCs of SPVec and other models on DTI predictions using DrugBank.

**Drug features**	**Drug** **dim**.	**Protein features**	**Protein dim**.	**ML method**	**AUC**	**References**
Drug structure information	2,216	AAC, DC^a^ and TC^b^	11,943	DNN	0.81	You et al., 2019^c^
Constitutional, topological and molecular descriptors, 2D autocorrelations, topological charge indices, eigenvalue-based indices	1,664	AAC; DC^a^; autocorrelation; Composition, Transition, Distribution descriptors; Quasi-sequence-order	1,080	RF	0.8950	Yu et al., 2012^c^
Constitutional, topological and geometrical descriptors	193	AAC; DC^a^; autocorrelation; composition, transition and distribution; quasi-sequence-order; amphiphilic pseudo-amino acid composition and total amino acid properties	1,260	DT RF	0.760 0.855	Ezzat et al., 2016
PubChem fingerprints indicating presence or absence of 881 known chemical substructures	881	Fingerprints of 876 different protein domains that are obtained from the Pfam database	876	EnsemDT	0.882	Ezzat et al., 2017
				RF	0.855	
SMILES2Vec	100	ProtVec	100	GBDT	0.9467	This work
				RF	0.9469	
				DNN	0.8637	

a DC, dipeptide composition;

b TC, tripeptide composition;

c *These models are trained on different versions of DrugBank, whose AUCs are only as references*.

### Evaluation of the Robustness of SPVec

**Figure 4:**
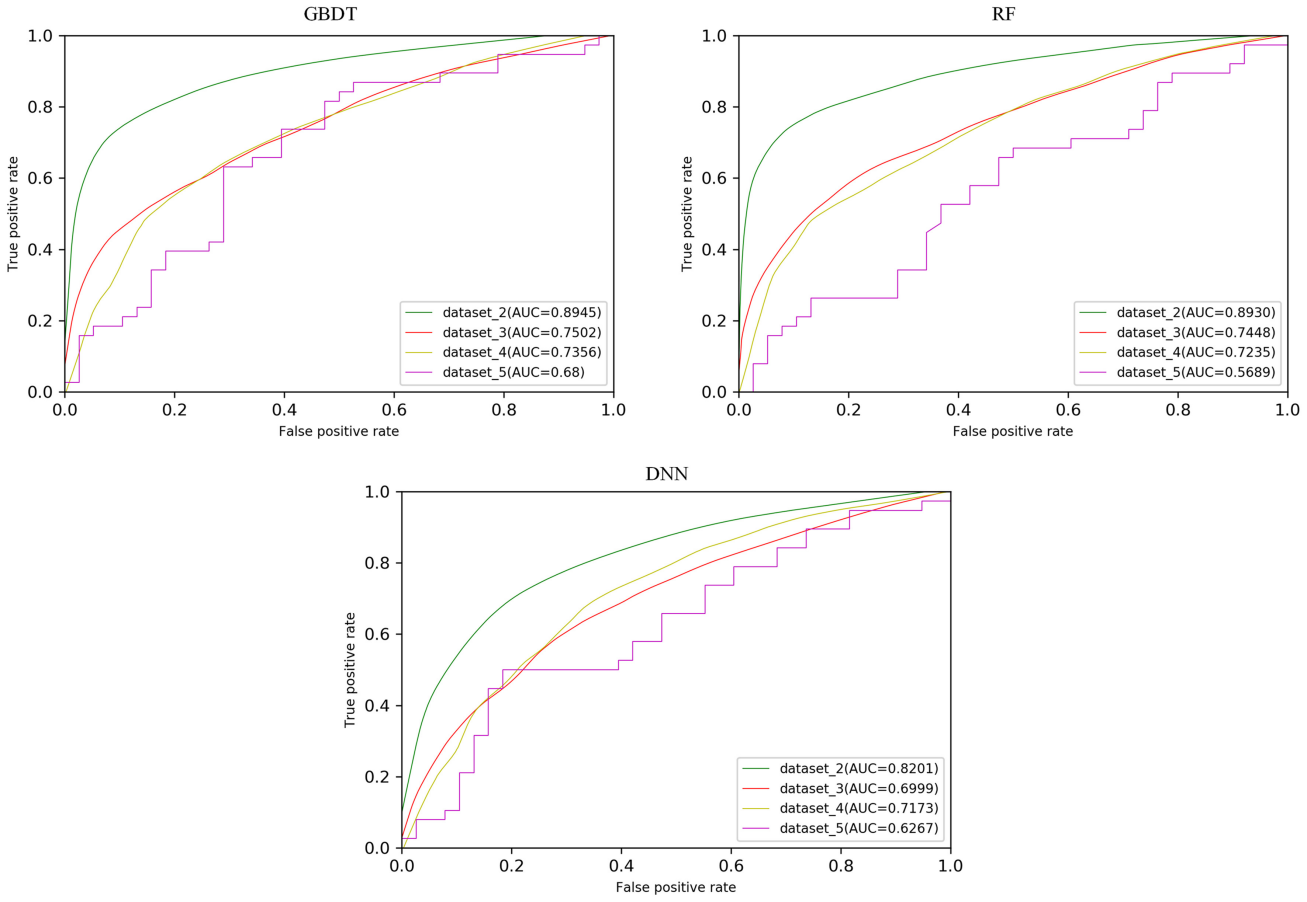
ROC curves with different models on the test sets obtained from DrugBank.

In order to test the robustness of SPVec in DTIs predictions, especially in the newly found interactions, five datasets were constructed from DrugBank, as described in section Datasets. We used dataset_1 as the training set to learn features and construct the ML models and then tested their performances on the datasets with new data. The classification performances on dataset_1 via 10 × 5-fold cross-validation and performances on independent test sets like dataset_1, dataset_2, and dataset_3 using GBDT, RF and DNN are summarized in Table 4 with their ROC curves shown in Figure 4. As in Table 4, the ML approaches equipped with the SPVec features got quite high AUC on the training set, which is similar to the results on BindingDB. Although DNN architecture was outperformed by the tree-based methods GBDT and RF in both cases, we would like to note the possibility that further fine-tuning might a little bit improve the prediction performance of the SPVec-DNN combination. Table 4 shows that SPVec performed satisfactorily on the test sets, suggesting acceptable generalization capacity and competitive performance of SPVec for the prediction of novel DTIs in drug repositioning or drug rediscovery, which is also suggested by the ROC curves in Figure 4. Among the four test sets, all three classifiers achieve highest AUC on dataset_2 to predict new interactions between old drugs and old targets, while the prediction results on the interactions with new drugs or new targets are much worse, which is extraordinary obvious in the ROC curves of dataset_5 in Figure 4. A possible explanation is that the newly found drugs or targets are not studied adequately and many potential DTIs between them have not been identified yet. Thus, the reduced accuracy of the data impairs the accuracy of the models. It is also worth noting that the negative sample was constructed by randomly selection from the unlabeled data, where the portion of unidentified potential positive DTI pairs may be even higher in smaller datasets. At last, the distributions of the new DTIs in the vector space of the test sets may be deviated from that of the training set, and impair the robustness of the models.

**Table 4:** Results of classification performance using three classifiers on datasets obtained from DrugBank, with the highest scores highlighted in the bold font.

**Dataset**	**Model**	**AUC**	**Accuracy**	**Precision**	**Recall**	**F1-score**
**Training set**	**10 × 5-fold cross-validation**					
Dataset_1	GBDT	0.9506	**0.9323**	**0.9456**	0.9367	**0.9343**
	RF	**0.9557**	0.9234	0.9378	**0.9369**	0.9337
	DNN	0.8952	0.8732	0.8345	0.8437	0.8654
**Test sets**	**Independent validation**					
	GBDT	**0.8945**	0.8628	**0.8747**	**0.8696**	**0.8637**
Dataset_2	RF	0.8930	**0.8753**	0.8645	0.8467	0.8555
	DNN	0.8201	0.8026	0.8138	0.8199	0.8144
	GBDT	**0.7502**	**0.7389**	**0.7340**	**0.7245**	**0.7333**
Dataset_3	RF	0.7448	0.7299	0.7198	0.7243	0.7230
	DNN	0.6999	0.6922	0.6825	0.6798	0.6832
	GBDT	**0.7356**	**0.7223**	**0.7167**	**0.7177**	**0.7201**
Dataset_4	RF	0.7235	0.7034	0.7108	0.7078	0.71
	DNN	0.7173	0.6899	0.6884	0.6896	0.6866
	GBDT	**0.68**	**0.6703**	**0.6679**	**0.6664**	**0.6688**
Dataset_5	RF	0.5689	0.5605	0.5398	0.5321	0.5411
	DNN	0.6267	0.6098	0.607	0.6122	0.6114

Particularly, the SPVec-GBDT method achieves the best performance among these three classifiers in DTIs predictions on the four test sets, with the AUCs as 0.8945, 0.7502, 0.7356, and 0.68, respectively. Although GBDT and RF showed similar results on the first four datasets, GBDT outperformed RF on dataset_5. This indicates that the SPVec-GBDT method may have better generalization capacity to achieve more robust prediction results, even for new drug-target pairs with limited or no interactions information, which may suggest that the SPVec-GBDT prediction model is possibly highly pertinent to the prediction of novel DTIs in drug repositioning.

### Prediction and Validation on Unidentified DTIs

Although the SPVec vectors with continuous values show competitive performance in the task of DTIs predictions, a dominant issue in the prediction of DTIs is that only confirmed positive interactions are deposited in the databases while those unlabeled interactions are unknown to be really positive or negative. For example, the newly found interactions in Drugbank might be thought negative in dataset_1. To further evaluate the validity of SPVec predictions on DTIs, the possibilities of all unlabeled DTIs in DrugBank dataset were evaluated using SPVec-GBDT and the top five ranked interactions were tested by external supporting evidences from several reference databases like PubChem (Wang et al., 2009), KEGG (Kanehisa and Goto, 2000), ChEMBL (Gaulton et al., 2017) and biomedical literatures. As a result, two of the top five predicted DTIs were confirmed by existing evidences (Table 5). The tyrosine-protein kinase Yes (Target ID: P07947, also known as Yes1) has been implicated as a potential therapeutic target in lots of cancers including breast cancers, melanomas, and rhabdomyosarcomas. Saracatinib (Drug ID: DB11805**)** was identified by Patel et al. (2013) as a potent Yes1 kinase inhibitor with the IC_50_ as low as 6.2nM. Our results also predicted the interaction between Pelitinib (Drug ID: DB05524) and membrane-associated tyrosine and threonine-specific cdc2-inhibitory kinase (Target ID: P42262) which was confirmed by PubChem database. Pelitinib (EKB-569) is a potent, low molecular weight, selective, and irreversible inhibitor of epidermal growth factor receptor (EGFR) in development as an anticancer agent, while membrane-associated tyrosine and threonine-specific cdc2-inhibitory kinase is the kinase domain of human myt1. These results demonstrate that the SPVec-DTIs model has highly useful pertinence for the prediction of novel DTIs.

**Table 5:** Top five novel DTIs predicted by SPVec-GBDT.

**Drug ID**	**Target ID**	**Drug name**	**Target name**	**Validation source**
DB11805	P07947	Saracatinib	The tyrosine-protein kinase Yes	Patel et al., 2013
DB09282	P42262	Molsidomine	Glutamate receptor 2	None
DB05524	Q99640	Pelitinib	Membrane-associated tyrosine and threonine-specific cdc2-inhibitory kinase	https://pubchem.ncbi.nlm.nih.gov/compound/6445562
DB03017	Q16620	Lauric acid	BDNF/NT-3 growth factors receptor	None
DB13165	P11362	Ripasudil	Fibroblast growth factor receptor 1	None

## Conclusion

Combining SMILES2Vec and ProtVec, SPVec could transfer SMILES strings of drug compounds and protein sequences into information-rich and lower-dimensional vectors automatically. Visualization of SPVec vectors nicely illustrates that the derived vectors from similar structures locate closely in the vector space, suggesting that they may implicitly reveals some important biophysical and biochemical patterns. Based on BindingDB and DrugBank database, SPVec vectors were fed into several state-of-art machine learning methods like GBDT, RF and DNN to train DTIs prediction models. The results using BindingDB have shown that the proposed models can achieve better prediction performance than manually extracted features like the combination of MACCS and AAC. Also, the results tested on DrugBank datasets indicated that our approach, especially SPVec-GBDT, can discover reliable DTIs in newly found drugs and targets, which might be beneficial for drug re-profiling. At last, all the unlabeled DTIs in DrugBank database was repredicted by the SPVec-GBDT model, and two of the top five predicted novel DTIs were confirmed by external evidences from other databases or biomedical literatures. In addition, SPVec vectors also have the advantages of automatic learning and lower dimensionality, which may significantly speed up training and reduces memory requirements, making it a highly potential method of feature representation for DTI predictions.

## Data Availability Statement

The datasets generated for this study can be found in the https://github.com/dqwei-lab/SPVec.

## Author Contributions

Y-FZ, QX, and D-QW made the conception and designed the study. Y-FZ and XW collected and organized the database. Y-FZ, AK, and YC performed the statistical analysis. QX and Y-FZ wrote the manuscript. XS contributed to part of the first draft of the manuscript. M-ZZ contributed to part of the manuscript. All authors contributed to manuscript revision, read and approved the submitted version.

### Conflict of Interest

The authors declare that the research was conducted in the absence of any commercial or financial relationships that could be construed as a potential conflict of interest.
