# Supplementary material for: SPVec: A Word2vec-Inspired Feature Representation Method for Drug-Target Interaction Prediction
Source: Front Chem. 2020 Jan 10;7:895. doi: 10.3389/fchem.2019.00895 (PMC6967417; doi:10.3389/fchem.2019.00895)
Supplement: Supplementary file 1 [file Table_1.docx]

# SPVec: A Word2vec-inspired feature representation method for Drug-Target Interaction Prediction

Yu-Fang Zhang^1^, Xiangeng Wang^1,2^, Aman Chandra Kaushik^1,3^, Yanyi Chu^1^, Xiaoqi Shan^1^, Ming-Zhu Zhao^4^, Qin Xu^1^* and Dong-Qing Wei^1^*

^1^State Key Laboratory of Microbial Metabolism, and SJTU-Yale Joint Center for Biostatistics and Data Science, School of Life Sciences and Biotechnology, and Joint Laboratory of International Cooperation in Metabolic and Developmental Sciences, Ministry of Education, Shanghai Jiao Tong University, Shanghai 200240, China

^2^Peng Cheng Laboratory, Shenzhen, Guangdong 518055, China

^3^Wuxi School of Medicine, Jiangnan University, Wuxi, Jiangsu 214122, China

^4^Instrumental Analysis Center, Shanghai Jiao Tong University, Shanghai 200240, China

* Correspondence: xuqin523@sjtu.edu.cn (Q.X.); dqwei@sjtu.edu.cn (D.-Q.W.)

***Keywords:*** drug-target interaction, representation learning, Word2vec, machine learning, feature embedding

## Supplementary Materials


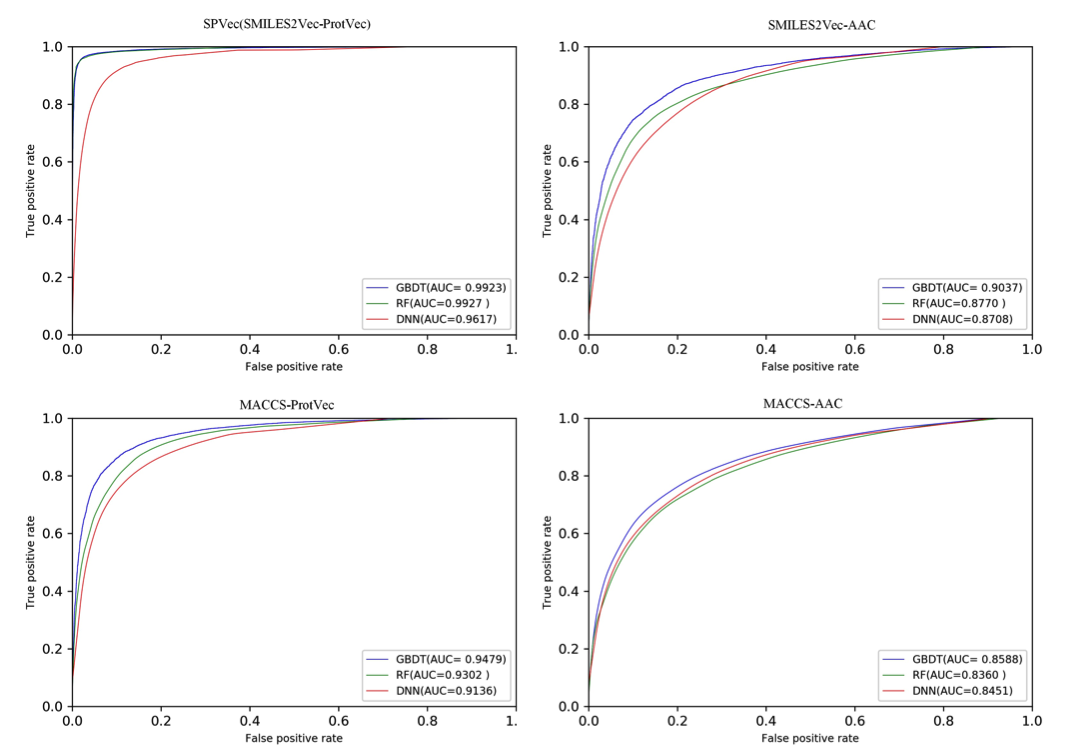


**Figure S1.** ROC curves of the four feature combinations using 10×5-fold cross-validation on BindingDB

**Table S1.** Results of classification performance using different versions of DrugBank datasets

| Durgbank Version | Released on | Model | AUC | Accuracy | Precision | Recall | F1-score |
| --- | --- | --- | --- | --- | --- | --- | --- |
| 4.3.0 | 2015-11-17 | GBDT | 0.9467 | 0.93 | 0.9389 | 0.9356 | 0.9377 |
|  |  | RF | 0.9469 | 0.9246 | 0.9397 | 0.9334 | 0.9369 |
|  |  | DNN | 0.8637 | 0.8578 | 0.8301 | 0.8423 | 0.8570 |
| 4.5.0 | 2016-04-20 | GBDT | 0.9506 | 0.9323 | 0.9456 | 0.9367 | 0.9343 |
|  |  | RF | 0.9557 | 0.9234 | 0.9378 | 0.9369 | 0.9337 |
|  |  | DNN | 0.8952 | 0.8732 | 0.8345 | 0.8437 | 0.8654 |

The version 4.5 added newly discovered dug-target interactions by ~22% than version 4.3. The performance of DNN improved a little bit, possibly because deep learning methods generally require large datasets. At the same time, the performance of GBDT and RF were not so sensitive to the size of the data set.
